# Supplementary material for: Automatic heliostat learning for in situ concentrating solar power plant metrology with differentiable ray tracing
Source: Nat Commun. 2024 Aug 14;15:6997. doi: 10.1038/s41467-024-51019-z (PMC11324880; doi:10.1038/s41467-024-51019-z)
Supplement: Supplementary file 1 — Supplementary Information [file 41467_2024_51019_MOESM1_ESM.pdf]

Supplementary Information: Automatic Heliostat Learning for In-situ Concentrating Solar Power Plant Metrology with Differentiable Ray Tracing

Dr. Max Pargmann,[1] Jan Ebert,[2, 3] Dr. Markus Götz,[2, 4] Dr. Daniel Maldonado Quinto,[1] Prof. Dr. Robert Pitz-Paal,[1, 5] and Dr. Stefan Kesselheim[2, 3]

[1] *Institute of Solar Research, German Aerospace Center (DLR)*
[2] *Helmholtz AI*
[3] *Jülich Supercomputing Centre, Research Institute Jülich (FZJ)*
[4] *Scientific Computing Centre (SCC), Karlsruhe Institute of Technology (KIT)*
[5] *Chair of Solar Technology, RWTH Aachen University*

(Dated: July 9, 2024)

HYPERPARAMETERS

The fitting procedure has been executed in a typical two-stage pipeline, i.e., training on parts of the data and evaluation of the generalization performance on a disjoint, held-out test set. The full set of default hyperparameters used during the fitting phase are shown in Table I. All parameters have been determined through empirical study.

TABLE I. Hyperparameters for the fitting phase of the differentiable ray tracer.

**Parameter**	**Value**
NURBS dimensions	7×7
NURBS spline degree	3
optimize control point z-dimension only	yes
optimization objective	L1
optimizer	Adam
learning rate	0.0001
Adam βs	[0.9, 0.999]
schedule base learning rate	$1 \cdot 10^{-8}$
learning rate schedule	cyclic [1]
schedule maximum learning rate	$8.3 \cdot 10^{-5}$
schedule momentum cycling	no
schedule policy	`triangular2`
cycle frequency	200 steps
# rays from sun per discrete heliostat point	150
latitude	50.92°
longitude	6.36°
receiver center	[0, -3.23, 35.89]
receiver plane normal	[0, 1, 0]
receiver plane width	8.63 m
receiver plane height	7.0 m
image resolution	64 px × 64 px
aim target	receiver center
heliostat field position	[13.2, 25.0, 1.795]
# discretization points	8,000
sun distribution	normal
sun distribution covariance	$4.3681 \cdot 10^{-6} \cdot \mathbb{I}$
sun distribution mean	[0, 0]
floating point precision	64 bit

TRAINING DATA

Figure 1 depicts the training data used for the comparative evaluation of the irradiance profile reconstruction used in the main manuscript. The data has been recorded in April of the same year. Noteworthy, the reconstructive performance is similar to the held-out test data, suggesting a reasonable generalization performance.

FULL LOSS FORMULATION

We present here the full formulation of the loss, including terms omitted from the main text.

$$L = \alpha_{\text{raw}} L_{\text{raw}} + \alpha_{\text{miss}} L_{\text{miss}} + \alpha_{\text{align}} L_{\text{align}} + \alpha_{\text{wd}} L_{\text{wd}}, \quad (1)$$

where

$$L_{\text{raw}} = \frac{1}{n_i \cdot n_j} \sum_{i,j} \left| E_{ij} - \hat{E}_{ij} \right|^{p_{\text{raw}}}, \quad (2)$$

with E_{ij} the irradiance of a ground truth image and $\hat{E}_{ij}$ the irradiance of an image obtained from ray tracing a learned heliostat, each at pixel position (i, j) of an image of size (n_i, n_j),

$$L_{\text{miss}} = \frac{1}{|X|^2} \sum_{\vec{x} \in X} \left| x_1 - \min\left(\max\left(x_1, -1\right), t_w + 1\right) \right|^{p_{\text{miss}}} + \left| x_2 - \min\left(\max\left(x_2, -1\right), t_h + 1\right) \right|^{p_{\text{miss}}}, \quad (3)$$

with X containing all rays intersecting an infinitely wide and high target plane, and t_w and t_h giving the actual target plane's width and height, respectively,

$$L_{\text{align}} = \frac{1}{|\vec{z}|} \sum_i |z_i - \hat{z}_i|^{p_{\text{align}}}, \quad (4)$$

with $\vec{z}$ and $\hat{\vec{z}}$ giving the ground truth and learned heliostat's alignment, respectively. Finally, the L1 weight decay term is given by

$$L_{\text{wd}} = \sum_{\vec{p} \in P} \sum_{v \in \vec{p}} |v|^{p_{\text{wd}}}, \quad (5)$$

where P contains all parameters to optimize in vector form.

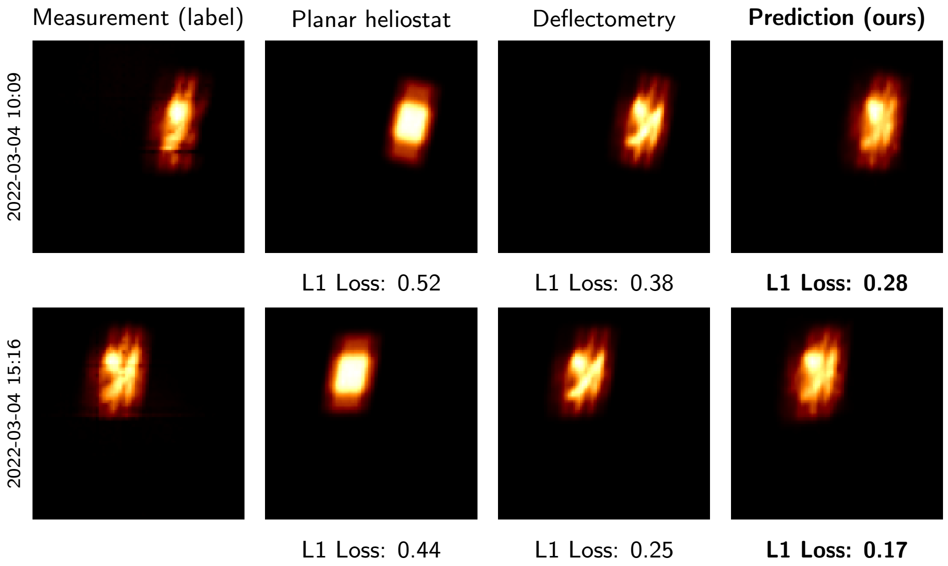

FIG. 1. Comparison of the irradiance profiles obtained from calibration images with naïve ray tracing of a planar heliostat, a deflectometry measurement, and the prediction of the differentiable ray tracer. Deviation between the measurement and each generated image is quantified using the L1 loss (↓, lower is better). The performance on training data only is shown.

COMPUTATIONAL ENVIRONMENT

We ran the experiments on the distributed-memory, parallel hybrid supercomputer *JUWELS Booster* [2]. Each of its 936 compute nodes is equipped with two 24-core AMD EPYC 7402 processors at 2.8 GHz base and 3.35 GHz maximum turbo frequency, 512 GB local memory, and 4 inter-node network cards Mellanox InfiniBand HDR interconnect with 200 Gbit/s per port. Each computational node is additionally equipped with four NVIDIA A100-40 GPUs with 40 GB memory connected via NVLink 3.

SOFTWARE ENVIRONMENT

The code used for the experiments was compiled with GCC version 11.3.0 and CUDA 11.7.64, and was run with Python 3.10.4, OpenBLAS 0.3.20, Intel oneAPI MKL 2022.1.0, PyTorch 1.12.0 [3], cuDNN 8.6.0.163. While we executed the code on a supercomputer to speed up parallel ablation experiments, there is no need for a large-scale setup during operation. Our optimization procedure runs in soft real-time on typical personal computers in CSPs.

APPLICATION TO LARGER FIELDS

The applicability of our method for 20 m to 400 m is highlighted in the main manuscript. To show that our method is also applicable for large fields, the predictions for 800 m to 1600 m are also shown here. Since the irradiance image is a convolution of the solar function and the heliostat surface function, the influence of the heliostat surface is correspondingly small at such distances and the flux density is dominated by the solar distribution function. Surfaces may be approximated with a significantly lower number of NURBS support points. The images shown in Figure 2 have been generated with 7×7 NURBS and trained on two images. The blurring of the focal spot described in Figure 4 occurs in the solar field not only due to the increased influence of the solar function but also because of the microscopic roughness of the mirror, potential soiling, and atmospheric scattering. In this study, a heliostat from the front row of the heliostat field was investigated, allowing these influences to be neglected. However, they must be considered in larger fields.

OPTICAL PROPERTIES

For a better understanding of our measurement setup, the optical parameters of the camera, the calibration target and the solar mirrors are summarized in Table II.
The heliostat surface utilized in this research is made of a solar mirror with a mean solar reflectivity of 94% (ISO9050, AM1.5). The glass surface has waviness according to DIN EN 572-1/-2. For this study, no reflectivity measurement of the exact mirror used in this publication was conducted, however Figure 3 shows a typical reflectivity for this kind of mirror measured in Jülich.
The calibration target is coated with a 25 µm Polyvinylidene fluoride (PVDF) varnish in the defined color 9010.

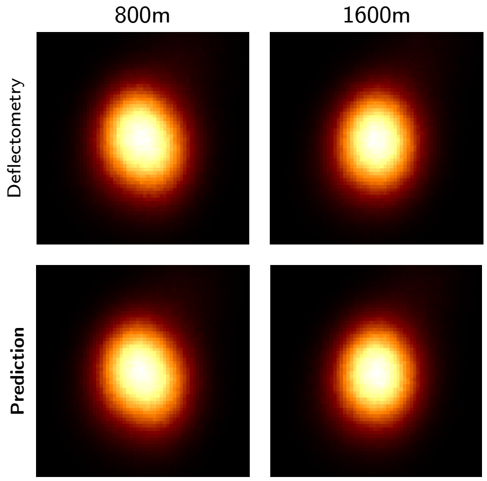

FIG. 2. Simulated and inferred irradiance profiles at large distances between heliostat and calibration target.

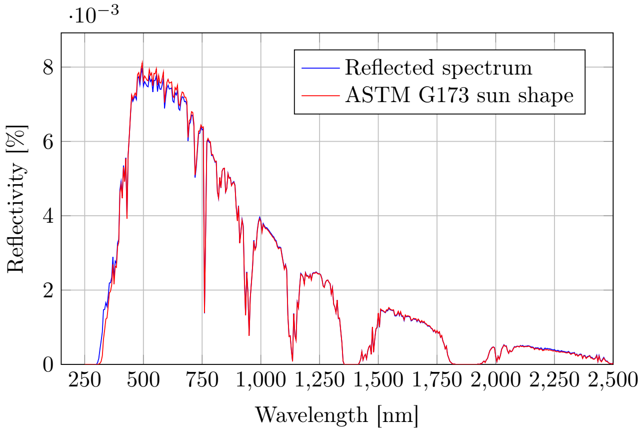

FIG. 3. Sun spectrum G173 and reflectivity measurement of a mirror at the solar tower power plant in Jülich. The absolute reflectivity is over 94%.

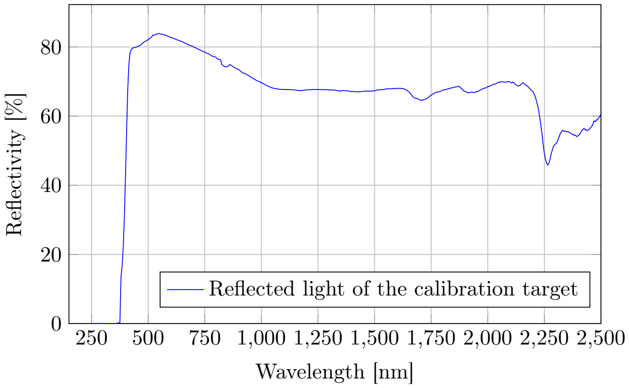

FIG. 4. Measured reflectance of one panel of the calibration target

Literature shows absorption values for different white colors in between 0.2 and 0.4 [4]. A more precise determination of the solar absorptance of the calibration target was made possible by determining the solar reflectance of a sample of the coated sheet metal in a laboratory using the Perkin Lambda 950 photospectrometer. The reflectance values for the visible light spectrum were determined in 5 nm steps and weighted using the solar spectrum. The entire measurement curve is shown in Figure 4.

Both the reflectivity of the target and the absorber were set as ideal in our simulation.

TABLE II. Environment and Measurement Parameters.

Parameter	Value
Atmospheric losses	None
Reflectivity mirror in simulation	100%
Reflectivity mirror at the solar tower	See Figure 3
Reflectivity of target in simulation	ideal white
Reflectivity target at the solar tower	See Figure 4
Camera	Axis Q1647-LE
Camera lens	AXIS Fujinon C/CS 8-80mm DC

[1] L. N. Smith, in *2017 IEEE winter conference on applications of computer vision (WACV)* (IEEE, 2017) pp. 464–472.

[2] S. Kesselheim, A. Herten, K. Krajsek, J. Ebert, J. Jitsev, M. Cherti, M. Langguth, B. Gong, S. Stadtler, A. Mozaffari, *et al.*, in *High Performance Computing: ISC High Performance Digital 2021 International Workshops, Frankfurt am Main, Germany, June 24–July 2, 2021, Revised Selected Papers 36* (Springer, 2021) pp. 453–468.

[3] A. Paszke, S. Gross, F. Massa, A. Lerer, J. Bradbury, G. Chanan, T. Killeen, Z. Lin, N. Gimelshein, L. Antiga, A. Desmaison, A. Kopf, E. Yang, Z. DeVito, M. Raison, A. Tejani, S. Chilamkurthy, B. Steiner, L. Fang, J. Bai, and S. Chintala, in *Advances in Neural Information Processing Systems*, Vol. 32 (Curran Associates, Inc., 2019).

[4] J. H. Henninger, *Solar Absorptance and Thermal Emittance of Some Common Spacecraft Thermal-Control Coatings*, NASA Reference Publication (RP) NASA-RP-1121, NAS 1.61:1121, REPT-84F0248 (NASA Goddard Space Flight Center, 1984) document ID: 19840015630, Accession Number: 84N23698, Acquisition Source: Legacy CDMS, Project: RTOP 845-17-07.
